# Supplementary material for: Psychosocial family-level mediators in the intergenerational transmission of trauma: Protocol for a systematic review and meta-analysis
Source: PLoS One. 2022 Nov 15;17(11):e0276753. doi: 10.1371/journal.pone.0276753 (PMC9665367; doi:10.1371/journal.pone.0276753)
Supplement: S1 Checklist — (DOCX) [file pone.0276753.s001.docx]

| Section and topic | No | Checklist item | Location in Manuscript |
| --- | --- | --- | --- |
| ADMINISTRATIVE INFORMATION | | |  |
| Title: |  |  |  |
| Identification | 1a | Identify the report as a protocol of a systematic review | Title |
| Update | 1b | If the protocol is for an update of a previous systematic review, identify as such | Not applicable |
| Registration | 2 | If registered, provide the name of the registry (such as PROSPERO) and registration number | MATERIALS AND METHODS: paragraph two |
| Authors: |  |  |  |
| Contact | 3a | Provide name, institutional affiliation, e-mail address of all protocol authors; provide physical mailing address of corresponding author | Title page |
| Contributions | 3b | Describe contributions of protocol authors and identify the guarantor of the review | AUTHORS’ CONTRIBUTIONS |
| Amendments | 4 | If the protocol represents an amendment of a previously completed or published protocol, identify as such and list changes; otherwise, state plan for documenting important protocol amendments | MATERIALS AND METHODS: paragraph two |
| Support: |  |  |  |
| Sources | 5a | Indicate sources of financial or other support for the review | FUNDING |
| Sponsor | 5b | Provide name for the review funder and/or sponsor | Not applicable |
| Role of sponsor or funder | 5c | Describe roles of funder(s), sponsor(s), and/or institution(s), if any, in developing the protocol | Not applicable |
| INTRODUCTION | | |  |
| Rationale | 6 | Describe the rationale for the review in the context of what is already known | BACKGROUND, paragraphs 6 and 7 |
| Objectives | 7 | Provide an explicit statement of the question(s) the review will address with reference to participants, interventions, comparators, and outcomes (PICO) | REVIEW QUESTIONS |
| METHODS | | |  |
| Eligibility criteria | 8 | Specify the study characteristics (such as PICO, study design, setting, time frame) and report characteristics (such as years considered, language, publication status) to be used as criteria for eligibility for the review | MATERIALS AND METHODS: Inclusion criteria |
| Information sources | 9 | Describe all intended information sources (such as electronic databases, contact with study authors, trial registers or other grey literature sources) with planned dates of coverage | MATERIALS AND METHODS: Protocol registration and timeline, Searches, and Source of evidence selection |
| Search strategy | 10 | Present draft of search strategy to be used for at least one electronic database, including planned limits, such that it could be repeated | Supplementary materials, eTables 3-8 |
| Study records: |  |  |  |
| Data management | 11a | Describe the mechanism(s) that will be used to manage records and data throughout the review | MATERIALS AND METHODS: Source of evidence selection, Data extraction |
| Selection process | 11b | State the process that will be used for selecting studies (such as two independent reviewers) through each phase of the review (that is, screening, eligibility and inclusion in meta-analysis) | MATERIALS AND METHODS: Source of evidence selection, Data extraction |
| Data collection process | 11c | Describe planned method of extracting data from reports (such as piloting forms, done independently, in duplicate), any processes for obtaining and confirming data from investigators | MATERIALS AND METHODS: Data extraction |
| Data items | 12 | List and define all variables for which data will be sought (such as PICO items, funding sources), any pre-planned data assumptions and simplifications | MATERIALS AND METHODS: Data extraction and eTable 9 |
| Outcomes and prioritization | 13 | List and define all outcomes for which data will be sought, including prioritization of main and additional outcomes, with rationale | Not applicable, as mediators and moderators are the variables of interest |
| Risk of bias in individual studies | 14 | Describe anticipated methods for assessing risk of bias of individual studies, including whether this will be done at the outcome or study level, or both; state how this information will be used in data synthesis | MATERIALS AND METHODS: Meta-analyses: Assessment of methodological quality |
| Data synthesis | 15a | Describe criteria under which study data will be quantitatively synthesised | MATERIALS AND METHODS: Meta-analyses, paragraph 1 |
|  | 15b | If data are appropriate for quantitative synthesis, describe planned summary measures, methods of handling data and methods of combining data from studies, including any planned exploration of consistency (such as I^2^, Kendall’s τ) | MATERIALS AND METHODS: Meta-analyses: Mediation meta-analyses, Moderation meta-analyses |
|  | 15c | Describe any proposed additional analyses (such as sensitivity or subgroup analyses, meta-regression) | MATERIALS AND METHODS: Meta-analyses: Mediation meta-analyses, Moderation meta-analyses |
|  | 15d | If quantitative synthesis is not appropriate, describe the type of summary planned | Not applicable |
| Meta-bias(es) | 16 | Specify any planned assessment of meta-bias(es) (such as publication bias across studies, selective reporting within studies) | MATERIALS AND METHODS: Meta-analyses: Publication bias |
| Confidence in cumulative evidence | 17 | Describe how the strength of the body of evidence will be assessed (such as GRADE) | MATERIALS AND METHODS: Assessing confidence in cumulative evidence |
