# Supplementary material for: Psychosocial family-level mediators in the intergenerational transmission of trauma: Protocol for a systematic review and meta-analysis
Source: PLoS One. 2022 Nov 15;17(11):e0276753. doi: 10.1371/journal.pone.0276753 (PMC9665367; doi:10.1371/journal.pone.0276753)
Supplement: S1 File — (PDF) [file pone.0276753.s002.pdf]

SUPPLEMENTARY MATERIALS

Search term	Yield
((intergenerational trauma.mp) or (transgenerational trauma.mp)) and ((systematic review.mp.) or (scoping review.mp))	27

Table 1a. Search strategy used to identify relevant systematic and scoping reviews in the Ovid (Medical Literature Analysis and Retrieval System Online (MEDLINE)® 1946 to February 03 2021, Embase 1974 to 2021 February 03, and APA PsycInfo 1806 to January Week 4 2021 databases. Date of search: 5 February 2021.

Search term	Yield
Intergenerational trauma	9

Table 1b. Search strategy used to identify reviews currently in progress in PROSPERO (https://www.crd.york.ac.uk/prospero) systematic review registration database. Date of search: 4 March 2021.

Search term	Yield
"intergenerational trauma"	2
"transgenerational trauma"	0

Table 1c. Search strategy used to identify reviews registered as an Open Science Framework preprint database (https://osf.io/preprints/). Date of search: 4 March 2021.

eTable 1. Search strategies used to identify review articles that highlighted psychosocial mediators or moderators in the intergenerational transmission of trauma. We also included papers from the first author's personal library identified during initial screening (N=14).

Reference	Review type	G1 and G2 population and trauma type restriction(s)	Generational scope	Number of quantitative studies that appear to measure psychosocial mediators and/or moderators on offspring mental health
Peer-reviewed publications				
Cerdeña et al., 2021(1)	Scoping review	>5% Latinx populations in the United States and Canada who experienced "trauma or violence"	G1 and G2	15 (7 longitudinal; 1 interventional; 7 cross-sectional)
Dashorst et al., 2019(2)	Scoping review	Holocaust survivors	G1 and G2	23 (all quantitative studies; design unspecified)
Dekel et al., 2008(3)	Literature review	Male combat veterans	G1 and G2	6 (design unspecified)
Der Sarkissian & Sharkey, 2021(4)	Scoping review	Armenian genocide survivors	G1, G2, and G3	Not reported
Flanagan et al., 2020(5)	Scoping review	Refugees with direct exposure to war-related trauma	G1 and G2	8 (2 longitudinal; 6 cross-sectional)
Galler & Rabinowitz, 2014(6)	Literature review	Parental early adversity and trauma	Unspecified; papers focused on G1 and G2	1 (cross-sectional)
Gone et al., 2019(7)	Scoping review	Historical trauma among indigenous populations	G1, G2, and G3	11 (design unspecified)
Kaitz et al., 2009(8)	Literature review	Parents traumatized by terror	G1 and G2	Not reported
Kellerman, 2001(9)	Literature review	Holocaust survivors	Unspecified	Not reported
Leen-Felder et al., 2013(10)	Scoping review	Parental post-traumatic stress	G1 and G2	17 (design unspecified)
Lehrner & Yehuda, 2018(11)	Literature review	Holocaust survivors	G1, G2, and G3	Not reported
Morris et al., 2012(12)	Systematic review and analysis of moderator effects in a meta-analysis	Parental and offspring post-traumatic stress symptoms	G1 and G2	32 (cross-sectional and longitudinal)
Pearrow & Cosgrove, 2009(13)	Literature review	Combat-related PTSD among military personnel	G1 and G2	Not reported

Plant et al., 2018(14)	Systematic review	Maternal childhood trauma	G1 and G2	12 (10 longitudinal; 2 cross-sectional)
Sangalang et al., 2017(15)	Scoping review	Refugees with trauma “collective in nature and affected a targeted group of people bound by a common cultural identity”	G1 and G2	16 (1 longitudinal; 15 cross-sectional)
Smallwood et al., 2021(16)	Scoping review	Historical trauma among indigenous populations	G1, G2, and G3	6 (3 longitudinal; 2 cross-sectional; 1 case-control)
Su et al., 2020(17)	Systematic review and moderation meta-analysis	Maternal childhood maltreatment	G1 and G2	12 (longitudinal)
Van Ee et al., 2016(18)	Scoping review	Caregivers with PTSD	G1 and G2	72 (design unspecified)
Grey literature				
Kandlur 2021(19)	Rapid scoping review	Historical trauma among Native American communities	Unspecified	No quantitative studies identified
Constantino-Pettit, 2019(20)	PROSPERO registration for a systematic review; meta-analytic approach not reported	Mothers with childhood trauma	G1 and G2	Unavailable
Khan & Rumesa, 2020(21)	Literature review, published as a conference abstract	Childhood trauma	G1 and G2	Not reported

eTable 2. Literature reviews of quantitative studies on potential psychosocial mediators and moderators for the intergenerational transmission of trauma.

Section and topic	No	Checklist item	Location in Manuscript
ADMINISTRATIVE INFORMATION			
Title:			
Identification	1a	Identify the report as a protocol of a systematic review	Title
Update	1b	If the protocol is for an update of a previous systematic review, identify as such	Not applicable
Registration	2	If registered, provide the name of the registry (such as PROSPERO) and registration number	MATERIALS AND METHODS: paragraph two
Authors:			
Contact	3a	Provide name, institutional affiliation, e-mail address of all protocol authors; provide physical mailing address of corresponding author	Title page
Contributions	3b	Describe contributions of protocol authors and identify the guarantor of the review	AUTHORS' CONTRIBUTIONS
Amendments	4	If the protocol represents an amendment of a previously completed or published protocol, identify as such and list changes; otherwise, state plan for documenting important protocol amendments	MATERIALS AND METHODS: paragraph two
Support:			
Sources	5a	Indicate sources of financial or other support for the review	FUNDING
Sponsor	5b	Provide name for the review funder and/or sponsor	Not applicable
Role of sponsor or funder	5c	Describe roles of funder(s), sponsor(s), and/or institution(s), if any, in developing the protocol	Not applicable
INTRODUCTION			
Rationale	6	Describe the rationale for the review in the context of what is already known	BACKGROUND, paragraphs 6 and 7
Objectives	7	Provide an explicit statement of the question(s) the review will address with reference to participants, interventions, comparators, and outcomes (PICO)	REVIEW QUESTIONS
METHODS			
Eligibility criteria	8	Specify the study characteristics (such as PICO, study design, setting, time frame) and report characteristics (such as years considered, language, publication status) to be used as criteria for eligibility for the review	MATERIALS AND METHODS: Inclusion criteria
Information sources	9	Describe all intended information sources (such as electronic databases, contact with study authors, trial registers or other grey literature sources) with planned dates of coverage	MATERIALS AND METHODS: Protocol registration and timeline, Searches, and Source of evidence selection
Search strategy	10	Present draft of search strategy to be used for at least one electronic database, including planned limits, such that it could be repeated	Supplementary materials, eTables 3-8
Study records:			
Data management	11a	Describe the mechanism(s) that will be used to manage records and data throughout the review	MATERIALS AND METHODS: Source of evidence selection, Data extraction

Selection process	11b	State the process that will be used for selecting studies (such as two independent reviewers) through each phase of the review (that is, screening, eligibility and inclusion in meta-analysis)	MATERIALS AND METHODS: Source of evidence selection, Data extraction
Data collection process	11c	Describe planned method of extracting data from reports (such as piloting forms, done independently, in duplicate), any processes for obtaining and confirming data from investigators	MATERIALS AND METHODS: Data extraction
Data items	12	List and define all variables for which data will be sought (such as PICO items, funding sources), any pre-planned data assumptions and simplifications	MATERIALS AND METHODS: Data extraction and eTable 9
Outcomes and prioritization	13	List and define all outcomes for which data will be sought, including prioritization of main and additional outcomes, with rationale	Not applicable, as mediators and moderators are the variables of interest
Risk of bias in individual studies	14	Describe anticipated methods for assessing risk of bias of individual studies, including whether this will be done at the outcome or study level, or both; state how this information will be used in data synthesis	MATERIALS AND METHODS: Meta-analyses: Assessment of methodological quality
Data synthesis	15a	Describe criteria under which study data will be quantitatively synthesised	MATERIALS AND METHODS: Meta-analyses, paragraph 1
	15b	If data are appropriate for quantitative synthesis, describe planned summary measures, methods of handling data and methods of combining data from studies, including any planned exploration of consistency (such as I^2, Kendall's τ)	MATERIALS AND METHODS: Meta-analyses: Mediation meta-analyses, Moderation meta-analyses
	15c	Describe any proposed additional analyses (such as sensitivity or subgroup analyses, meta-regression)	MATERIALS AND METHODS: Meta-analyses: Mediation meta-analyses, Moderation meta-analyses
	15d	If quantitative synthesis is not appropriate, describe the type of summary planned	Not applicable
Meta-bias(es)	16	Specify any planned assessment of meta-bias(es) (such as publication bias across studies, selective reporting within studies)	MATERIALS AND METHODS: Meta-analyses: Publication bias
Confidence in cumulative evidence	17	Describe how the strength of the body of evidence will be assessed (such as GRADE)	MATERIALS AND METHODS: Assessing confidence in cumulative evidence

eTable 3. PRISMA-P (Preferred Reporting Items for Systematic review and Meta-Analysis Protocols) 2015 checklist (22).

Search term	
1	(intergeneration* or transgeneration* or multigeneration* or crossgeneration* or generation* or secondary or secondarily or tertiary).mp.[intergenerational concept]
2	intergenerational relations/ or exp adult survivors of child adverse events/ or exp parent-child relations/ [intergenerational concept]
3	(paternal* or maternal* or parent* or grandparent* or grandmother* or grandfather* or famil* or mother* or father* or child* or grandchild* or granddaughter* or grandson* or daughter* or son* or intergenerational* or transgenerational* or multigenerational* or generation* or caregiver* or mother-child).mp. [family setting]
4	((intergeneration* or transgeneration* or multigeneration* or crossgeneration* or generation* or secondary or secondarily or tertiary or historical or vicarious or empathetic or legacy or inherited or maternal or paternal or parent* or mother* or father*) and (trauma* or PTSD or posttrauma* or psychotrauma* or abus* or maltreat* or mistreat* or adverse childhood or ACE or ACEs or childhood adversity)).ti. [trauma concept]
5	((intergeneration* or transgeneration* or multigeneration* or crossgeneration* or generation* or secondary or secondarily or tertiary or historical or vicarious or empathetic or legacy or inherited or maternal or paternal or parent* or mother* or father*) and (trauma* or PTSD or posttrauma* or psychotrauma* or abus* or maltreat* or mistreat* or adverse childhood or ACE or ACEs or childhood adversity)).kf. [trauma concept]
6	((intergeneration* or transgeneration* or multigeneration* or crossgeneration* or generation* or secondary or secondarily or tertiary or historical or vicarious or empathetic or legacy or inherited or maternal or paternal or parent* or mother* or father*) adj4 (trauma* or PTSD or posttrauma* or psychotrauma* or abus* or maltreat* or mistreat* or adverse childhood or ACE or ACEs or childhood adversity)).mp. [trauma concept]
7	(1 or 2) and 3 and (4 or 5 or 6)

eTable 4. Final search strategy to identify epidemiological studies assessing the intergenerational transmission of trauma at the family- and parent-level in the Ovid Medical Literature Analysis and Retrieval System Online (MEDLINE)® 1946 to Present database.

Search term	
1	(intergeneration* or transgeneration* or multigeneration* or crossgeneration* or generation* or secondary or secondarily or tertiary).mp.
2	intergenerational relations/ or transgenerational patterns/
3	(paternal* or maternal* or parent* or grandparent* or grandmother* or grandfather* or famil* or mother* or father* or child* or grandchild* or granddaughter* or grandson* or daughter* or son* or intergenerational* or transgenerational* or multigenerational* or generation* or caregiver* or mother-child).mp. [family setting]
4	((intergeneration* or transgeneration* or multigeneration* or crossgeneration* or generation* or secondary or secondarily or tertiary or historical or vicarious or empathetic or legacy or inherited or maternal or paternal or parent* or mother* or father*) and (trauma* or PTSD or posttrauma* or psychotrauma* or abus* or maltreat* or mistreat* or adverse childhood or ACE or ACEs or childhood adversity)).ti,id.
5	((intergeneration* or transgeneration* or multigeneration* or crossgeneration* or generation* or secondary or secondarily or tertiary or historical or vicarious or empathetic or legacy or inherited or maternal or paternal or parent* or mother* or father*) adj4 (trauma* or PTSD or posttrauma* or psychotrauma* or abus* or maltreat* or mistreat* or adverse childhood or ACE or ACEs or childhood adversity)).mp.
6	transgenerational patterns/
7	trauma/ or emotional trauma/ or posttraumatic growth/ or posttraumatic stress/ or traumatic loss/ or complex ptsd/ or exp posttraumatic stress disorder/ or exp "stress and trauma related disorders"/
8	6 and 7
9	(1 or 2) and 3 and (4 or 5 or 8)

eTable 5. Final search strategy to identify epidemiological studies assessing the intergenerational transmission of trauma at the family- and parent-level in the Ovid PsycINFO® 1946 to Present database.

Search term	
1	(MAINSUBJECT.EXACT("Intergenerational Effects") OR NOFT(intergeneration* or transgeneration* or multigeneration* or crossgeneration* or generation* or secondary or secondarily or tertiary)) AND (NOFT(paternal* or maternal* or parent* or grandparent* or grandmother* or grandfather* or famil* or mother* or father* or child* or grandchild* or granddaughter* or grandson* or daughter* or son* or intergenerational* or transgenerational* or multigenerational* or generation* or caregiver* or "mother-child") OR MAINSUBJECT.EXACT.EXPLODE("Family Members"))

eTable 6. Final search strategy to identify epidemiological studies assessing the intergenerational transmission of trauma at the family- and parent-level in PTSDpubs database.

Search term	
1	(TITLE-ABS-KEY (intergeneration* OR transgeneration* OR multigeneration* OR crossgeneration* OR generation* OR secondary OR secondarily OR tertiary)) AND (TITLE-ABS-KEY (paternal* OR maternal* OR parent* OR grandparent* OR grandmother* OR grandfather* OR famil* OR mother* OR father* OR child* OR grandchild* OR granddaughter* OR grandson* OR daughter* OR son* OR intergenerational* OR transgenerational* OR multigenerational* OR generation* OR caregiver* OR mother-child)) AND ((TITLE ((intergeneration* OR transgeneration* OR multigeneration* OR crossgeneration* OR generation* OR secondary OR secondarily OR tertiary OR historical OR vicarious OR empathetic OR legacy OR inherited OR maternal OR paternal OR parent* OR mother* OR father*) AND (trauma* OR ptsd OR posttrauma* OR psychotrauma* OR abus* OR maltreat* OR mistreat* OR "adverse childhood" OR ace OR aces OR "childhood adversity"))) OR (TITLE-ABS-KEY ((intergeneration* OR transgeneration* OR multigeneration* OR crossgeneration* OR generation* OR secondary OR secondarily OR tertiary OR historical OR vicarious OR empathetic OR legacy OR inherited OR maternal OR paternal OR parent* OR mother* OR father*) W/4 (trauma* OR ptsd OR posttrauma* OR psychotrauma* OR abus* OR maltreat* OR mistreat* OR "adverse childhood" OR ace OR aces OR "childhood adversity")))) AND (LIMIT-TO (DOCTYPE , "cp"))

eTable 7. Final search strategy to identify epidemiological studies assessing the intergenerational transmission of trauma at the family- and parent-level in the Scopus database.

Search term	
1	(ti((intergeneration* OR transgeneration* OR multigeneration* OR crossgeneration* OR generation* OR secondary OR secondarily OR tertiary OR historical OR vicarious OR empathetic OR legacy OR inherited OR maternal OR paternal OR parent* OR mother* OR father*) AND (trauma* OR PTSD OR posttrauma* OR psychotrauma*)) OR ab(((intergeneration* OR transgeneration* OR multigeneration* OR crossgeneration* OR generation* OR secondary OR secondarily OR tertiary OR historical OR vicarious OR empathetic OR legacy OR inherited OR maternal OR paternal OR parent* OR mother* OR father*) NEAR/2 (trauma* OR PTSD OR posttrauma* OR psychotrauma*)))) AND (ti(paternal* or maternal* or parent* or grandparent* or grandmother* or grandfather* or famil* or mother* or father* or child* or grandchild* or granddaughter* or grandson* or daughter* or son* or intergenerational* or transgenerational* or multigenerational* or generation* or caregiver* or mother-child) or ab(paternal* or maternal* or parent* or grandparent* or grandmother* or grandfather* or famil* or mother* or father* or child* or grandchild* or granddaughter* or grandson* or daughter* or son* or intergenerational* or transgenerational* or multigenerational* or generation* or caregiver* or mother-child)) AND noft(intergeneration* or transgeneration* or multigeneration* or crossgeneration* or generation* or secondary or secondarily or tertiary)
2	Limit to doctoral dissertations

eTable 8. Final search strategy to identify epidemiological studies assessing the intergenerational transmission of trauma at the family- and parent-level in the ProQuest Dissertations and Theses database.

Category	Extracted data
Basic study information	1. Study design (cross-sectional, retrospective cohort, prospective cohort, or interventional)<br>2. Geographic location<br>3. Study setting (clinic, community, other)<br>4. Funding source<br>5. Sample size at baseline<br>1. Gender distribution<br>2. Mean and standard deviation of age (if unreported, age range)<br>6. Theoretical framework of intergenerational trauma (for example: attachment theory, family systems theory, family functioning theory, etc.).<br>7. Study definition of intergenerational transmission of trauma
Population characteristics	8. Generations observed (G1, G2, G3) and relationships observed (G1 to G2; G1 to G3; G2 to G3)<br>9. Information on G1 traumatic event:<br>1. Reported definition of G1 trauma<br>2. Discrete or continual event (if continual, information on frequency and time range)<br>3. Age of occurrence<br>4. Measurement tool used to measure traumatic event
Exposure, mediator, moderator, and outcome information	10. Mediating factors, for each eligible factor reported:<br>1. Level of mediation (whether G1 to G2; G1 to G3; G2 to G3 transmission)<br>2. Description of factor<br>3. What path is being mediated<br>4. Measurement tool(s)<br>5. Timing of measurement(s) in relation to child's age<br>6. Proportion of sample that experienced mediator versus comparator<br>7. Impact on G2 or G3 psychological symptoms/functioning:<br>1. Description of outcome(s)<br>2. Measurement tool(s) and results<br>3. Timing of measurement(s) in relation to child's age<br>8. If mediation analyses performed:<br>1. Sample size in analysis, analysis method (example: Baron and Kenny, structural equation modeling, etc.), standardized regression coefficient(s) for direct and indirect effects, significance test (example: Sobel), respective statistical significance level(s), covariates adjusted for, correlation matrix.<br>11. Moderating factors, for each eligible factor reported:<br>9. Level of moderation (whether G1 to G2; G1 to G3; G2 to G3 transmission)<br>10. Description of factor<br>11. What path is being moderated<br>12. Measurement tool(s)<br>13. Timing of measurement(s) in relation to child's age<br>14. Proportion of sample that experienced moderator/breakdown of levels<br>15. Impact on F1 or F2 psychological symptoms/functioning:<br>1. Description of outcome(s)<br>2. Measurement tool(s) and results<br>3. Timing of measurement(s) in relation to child's age<br>16. If statistical model performed: sample size in analysis, analytic method, effect estimate(s) and respective statistical significance level(s), adjusted covariates

eTable 9. Draft data extraction items.

Studies and reference(s)	Study design	Population and study setting	N at baseline	Age and gender distribution	Concept of G1 trauma (measure)	Mediating factors (measure, timepoints, effect estimates and significance levels; covariate adjustment)	Moderating factors (measure, timepoints, effect estimates and significance levels; covariate adjustment)	G2 mental health outcomes (measure, timepoints)
Intergenerational transmission from G1 to G2								
Study A…								
Study B…								
Intergenerational transmission from G2 to G3								
Study C…								
Study D…								
Intergenerational transmission from G1 to G3								
Study E…								

eTable 10. Draft table skeleton to provide overview of included studies and their respective results on mediators and/or moderators, sub-divided by generational transmission type.

Category	Psychosocial factor	Definitions, measurement tools reported and in which studies
Intergenerational transmission from G1 to G2		
G2 individual characteristics	Perceived transmission of trauma burden	
G1 individual characteristics	Severity of parental symptomatology and mental distress	
G1 relational characteristics	Parenting and attachment quality	
	Maladaptive parenting styles	
	Harsh parenting	
	Diminished parental emotional availability	
	Child maltreatment	
Family-level factors	Family structure	
	Decreased family functioning	
	Accumulation of family stressors	
	Dysfunctional intra-family communication styles	
	Environmental factors	
Intergenerational transmission from G2 to G3		
….		
Intergenerational transmission from G1 to G3		
….		

eTable 11. Draft table skeleton to present consolidated mediation constructs. Examples were inspired from factors in recent scoping reviews in anticipation of findings (1, 2, 5, 15, 21).

REFERENCES

1. Cerdeña JP, Rivera LM, Spak JM. Intergenerational trauma in Latinxs: A scoping review. Social Science & Medicine. 2021;270:113662.
2. Dashorst P, Mooren TM, Kleber RJ, de Jong PJ, Huntjens RJC. Intergenerational consequences of the Holocaust on offspring mental health: a systematic review of associated factors and mechanisms. Eur J Psychotraumatol. 2019;10(1):1654065-.
3. Dekel R, Goldblatt H. Is there intergenerational transmission of trauma? The case of combat veterans' children. Am J Orthopsychiatry. 2008;78(3):281-9.
4. Der Sarkissian A, Sharkey JD. Transgenerational Trauma and Mental Health Needs among Armenian Genocide Descendants. International journal of environmental research and public health. 2021;18(19):10554.
5. Flanagan N, Travers A, Vallières F, Hansen M, Halpin R, Sheaf G, et al. Crossing borders: a systematic review identifying potential mechanisms of intergenerational trauma transmission in asylum-seeking and refugee families. Eur J Psychotraumatol. 2020;11(1):1790283-83.
6. Galler J, Rabinowitz DG. The intergenerational effects of early adversity. Prog Mol Biol Transl Sci. 2014;128:177-98.
7. Gone JP, Hartmann WE, Pomerville A, Wendt DC, Klem SH, Burrage RL. The impact of historical trauma on health outcomes for indigenous populations in the USA and Canada: A systematic review. American Psychologist. 2019;74(1):20-35.
8. Kaitz M, Levy M, Ebstein R, Faraone SV, Mankuta D. The intergenerational effects of trauma from terror: A real possibility. Infant Mental Health Journal. 2009;30(2):158-79.
9. Kellermann NP. Transmission of Holocaust trauma-An integrative view. Psychiatry: Interpersonal and Biological Processes. 2001;64(3):256-67.
10. Leen-Feldner EW, Feldner MT, Knapp A, Bunaciu L, Blumenthal H, Amstadter AB. Offspring psychological and biological correlates of parental posttraumatic stress: Review of the literature and research agenda. Clinical Psychology Review. 2013;33(8):1106-33.
11. Lehrner A, Yehuda R. Trauma across generations and paths to adaptation and resilience. Psychological Trauma: Theory, Research, Practice, and Policy. 2018;10(1):22-9.
12. Morris A, Gabert-Quillen C, Delahanty D. The Association Between Parent PTSD/Depression Symptoms and Child PTSD Symptoms: A Meta-Analysis. Journal of Pediatric Psychology. 2012;37(10):1076-88.
13. Pearrow M, Cosgrove L. The Aftermath of Combat-Related PTSD: Toward an Understanding of Transgenerational Trauma. Communication Disorders Quarterly. 2008;30(2):77-82.
14. Plant DT, Pawlby S, Pariante CM, Jones FW. When one childhood meets another – maternal childhood trauma and offspring child psychopathology: A systematic review. Clinical Child Psychology and Psychiatry. 2017;23(3):483-500.
15. Sangalang CC, Vang C. Intergenerational Trauma in Refugee Families: A Systematic Review. J Immigr Minor Health. 2017;19(3):745-54.
16. Smallwood R, Woods C, Power T, Usher K. Understanding the Impact of Historical Trauma Due to Colonization on the Health and Well-Being of Indigenous Young Peoples: A Systematic Scoping Review. Journal of Transcultural Nursing. 2020;32(1):59-68.
17. Su Y, D'Arcy C, Meng X. Intergenerational Effect of Maternal Childhood Maltreatment on Next Generation's Vulnerability to Psychopathology: A Systematic Review With Meta-Analysis. Trauma, violence & abuse. 2020:1524838020933870.
18. van Ee E, Kleber RJ, Jongmans MJ. Relational Patterns Between Caregivers With PTSD and Their Nonexposed Children: A Review. Trauma, Violence, & Abuse. 2015;17(2):186-203.

19. Kandlur R. Psychosocial Mechanisms of Historical Trauma Transmission in Native American Communities: A Rapid Scoping Review: Yale University; 2021.
20. Anna Constantino-Pettit. Childhood trauma as a social determinant of health across generations: a systematic review of the biological and environmental mechanisms of intergenerational trauma 2019 [Available from: https://www.crd.york.ac.uk/prospero/display_record.php?RecordID=146949.
21. Khan M, Rumesa F, editors. Parental Trauma and Its Effects on Child Psychopathology: A Look at the Literature. 2020 Virtual Meeting; 2020: AACAP.
22. Moher D, Shamseer L, Clarke M, Ghersi D, Liberati A, Petticrew M, et al. Preferred reporting items for systematic review and meta-analysis protocols (PRISMA-P) 2015 statement. Systematic reviews. 2015;4(1):1-9.
23. Lee H, Hübscher M, Moseley GL, Kamper SJ, Traeger AC, Mansell G, et al. How does pain lead to disability? A systematic review and meta-analysis of mediation studies in people with back and neck pain. Pain. 2015;156(6):988-97.
