# Supplementary material for: Psychosocial family-level mediators in the intergenerational transmission of trauma: Protocol for a systematic review and meta-analysis
Source: PLoS One. 2022 Nov 15;17(11):e0276753. doi: 10.1371/journal.pone.0276753 (PMC9665367; doi:10.1371/journal.pone.0276753)
Supplement: Attachment — Submitted filename: Response To Reviewers.docx [file pone.0276753.s003.docx]

|  | Reviewer Comment | Authors’ Response |
| --- | --- | --- |
| General | | |
| 1 | Please ensure that your manuscript meets PLOS ONE's style requirements, including those for file naming. | Addressed. See referencing formatting changes throughout. |
| 2 | Please include captions for your Supporting Information files at the end of your manuscript, and update any in-text citations to match accordingly. Please see our Supporting Information guidelines for more information: http://journals.plos.org/plosone/s/supporting-information. | We have added in a Supporting Information subheading (page 16). We also uploaded the final PDF version of this document to the resubmission. |
| 3 | We note that this manuscript is a systematic review or meta-analysis; our author guidelines therefore require that you use PRISMA guidance to help improve reporting quality of this type of study. Please upload copies of the completed PRISMA checklist as Supporting Information with a file name “PRISMA checklist”. | We have included the PRISMA-P reporting guideline for systematic review protocols in the Supplementary Materials (S1 eTable 3). |
| Reviewer #1 | | |
| 4 | Goals:  • Meta-analyses of epidemiological research of causal mechanisms across a diversity of family level mediators and moderators that play a role in the intergenerational transmission across diverse trauma-types. | No comment. |
| 5 | Strengths  • This is a very comprehensive proposal for a systematic review and meta-analysis that would be a much-needed contribution to the literature.  • Study proposes to use well-established rigorous methodology/ framework: Cochrane Handbook for Systematic Reviews and Joanna Briggs Institute scoping review methods manual and addressing publication bias through forward citation chaining and including conference abstracts and dissertations.  • Inclusion of a broad range of psychosocial factors that might be mediators or moderators (e.g., trauma related mental health functioning, other psychological factors, interpersonal factors or social factors) between G1 exposure and G2 or G3 outcomes.  • Study will produce directed acyclic graph i.e. causal diagram(s) of identified mediators and moderators of intergenerational transmission of trauma. | Thank you for these comments about the strengths of our manuscript. |
| 6 | Minor suggestions for improvement:  The term "unresolved trauma" in the discussion section of the abstract and again towards the end of the comes as a surprise and appears unrelated to the Introduction section where the Phrase "intergenerational transmission of trauma" and its familial mediators are noted. In fact this term is not used in the rest of the manuscript until the conclusion. Rephrasing to be consistent across sections or defining the term clearly at the outset would be helpful. | Thank you for this comment. We agree that this was unclear in our original submission that that the text could be strengthened with clarification or standardization. To address this, we changed all references of “unresolved trauma” to “the psychological impacts of trauma” (see changes in the Abstract and Conclusions sections). |
| Reviewer #2 | | |
| 7 | The investigators present a protocol for a systematic review to identify factors associated with intergenerational transmission of trauma.  The background is well written and covers the literature on the topic, the knowledge gap, and the need for this systematic review. | Thank you. |
| 8 | The methodology is sound and justified. | Thank you. |
| 9 | I have the following comments.  1. Duplicate data extraction should not be based on feasibility, but a matter of principle to reduce errors. Single extraction and quality control is also acceptable. | Thank you for your comment. We have modified the language to reflect that data extraction will be performed in single alongside quality assurance checks (page 11). |
| 10 | 2. Dissertations are not generally considered to be peer-reviewed and may not be an appropriate source of information. | Thank you for raising this important concern. We agree that there is risk that there could be lower methodological quality of dissertations compared to the peer-reviewed publications; however, following the advice of our medical research librarian, and as reported in the literature (see Paez 2017 below), we feel it would be useful to include dissertations, given that it would mitigate publication bias and increase our yield to better support the likelihood of conducting mediation and moderation meta-analyses. This also seems to be the best course of action after preliminary screening, as nearly half of the included articles are projected to be dissertations. Research also suggests that the addition of dissertations would likely have little impact on meta-analytic results, though would be important to include in ‘situations where there are few relevant studies’, which we feel applies to this review where the bulk of the included studies would most likely be dissertations (Hartling et al, 2017; below).  To balance this limitation, however, we will be sure to evaluate all evidence using a critical appraisal tool, as discussed, and would highlight the quality of evidence in the results and discussion in the published reviews. We also plan to run sensitivity analyses prior to publication to assess whether dissertations and other forms of grey literature in the final sample impacted the pooled effect estimates; we will report these results in the final publication(s).  References:  Hartling L, Featherstone R, Nuspl M, Shave K, Dryden DM, Vandermeer B. Grey literature in systematic reviews: a cross-sectional study of the contribution of non-English reports, unpublished studies and dissertations to the results of meta-analyses in child-relevant reviews. BMC Medical Research Methodology. 2017;17(1):64.  Paez A. Grey literature: An important resource in systematic reviews. J Evid Based Med. 2017. |
| 11 | 3. It is an excellent idea to consult experts regarding the statistical analysis plan. It is even better to use this advise to develop the protocol. While there are many unknowns, it would be helpful to present some decision rules in this regard. For example, how much data do you need to decide about statistical pooling or not. Two statements highlight this issue:  “We will conduct meta-analyses following guidelines reported in the literature (29) that would be most suitable to the methods reported in our sample of included studies” and “We will perform moderation meta-analyses using standard statistical methods. (30)” | Thank you for this suggestion.  To address this, we have added more details with respect to the statistical analysis plan and decision rules to determine which studies would be eligible for the meta-analyses (see pages 13 and 14; under sections: Mediation meta-analyses and Moderation meta-analyses).  In order to best address this, we also added our biostatistician colleague as a co-author on this protocol, given that this protocol now includes an expanded statistical plan (Dr. Veronika Shabanova, who has also approved the changes in this resubmission). |
| 12 | Moderation meta-analyses are not standard and should be described in more detail. | Thank you for this suggestion. We have added in two additional sentences on our statistical analysis plan for the moderation meta-analyses on page 13. |
| 13 | This is also apparent in the abstract: “Findings will be presented using narrative syntheses, descriptive analyses, mediation meta-analyses, moderating meta analyses, and causal diagram(s), where possible.” There is no indication of how you will decide which approach to use. These decisions should not be made after seeing the data. | Thank you for bringing this gap to our attention; we agree, more detail was needed. To address this, we added more information under the section “Presentation of results” (pages 14-15). |
| 14 | 4. Another issue is the evaluation of study quality. The authors state that they will use the tools applied in other mediation meta-analyses. This is not appropriate. The tool must be stated and described, with details on the items assessed and how the information is interpreted. | Thank you for highlighting this gap for the reader; we agree. To address this concern, we made clear that the citation to the original paper that developed and reported this tool (page 14). Although we would also be very open to reporting the tool questions themselves in the Supplementary Materials, we do not want to infringe on the tool copyright from the original authors. We also added in a sentence explaining that we chose this tool because, to our knowledge, there are no other tools available to evaluate the quality of mediation studies. |
| 15 | 5. It would not be appropriate to use separate tools to assess study quality if this is not linked to the purpose of the SR. For example, an RCT may have high methodological quality (low risk of bias) but have other flaws that make it a poor study for evaluating moderation. The QUIPS tool can be used to assess risk of bias in prognosis studies, irrespective of the study design. If there is an equivalent tool for mediation studies, it should be the primary approach for ascertaining the study quality. | Thank you for this suggestion; we agree, the critical appraisal tool should best reflect the goal of the review. Building on your suggestion, we will use the mediation-specific tool to assess mediation paths; and we will use the study-level JBI tools to assess quality of moderation paths (as, to our knowledge, there is no tool available to specifically evaluate the quality of moderation studies).  Thank you for also directing us to the QUIPS tool. Reviewing this tool, it seems that it also might not be the best fit for moderation studies. For feasibility, too, we feel that the JBI tool would be better suited to evaluate moderation in lieu of a specific tool designed for this, as it is substantially shorter than the QUIPS tool and likely easier to attain higher inter-rater reliability between extractors compared to QUIPS, which appears to be more qualitative and subjective in nature. We do, however, acknowledge that the moderation meta-analyses and narrative synthesis that arise from this review will be limited applying the JBI tool, given that this tool would examine study-level quality rather than the examination of the specific moderation effect. |
| 16 | 6. Please provide details on how you will create a funnel plot to assess publication bias given the nature of your outcomes and data. Will you create one for each moderator/mediator? | Thank you for this suggestion. We clarified this further to explain that we aim to assess publication bias for each meta-analysis for each mediating construct (page 14). For the moderation meta-analyses, we do not plan on developing funnel plots for the moderating constructs, as we anticipate that there would only be such a small sample of included studies that would make it into our individual patient-level meta-analysis, and hence, we do not anticipate having enough information to develop funnel plots for this. |
| 17 | 7. There is no information on Assessing confidence in cumulative evidence. The sentence under this section is not related to confidence in cumulative evidence. GRADE can be used for a variety of evidence types including narrative synthesis (GRADE CERQAL). | Thank you for this suggestion; we added GRADE to this section to specify (page 15). |
| 18 | Overall, the rational for this project is sound, but there is insufficient detail for the work to be implemented and too many decisions will be made based on the nature of the data. This is an important concern. | Thank you for addressing this concern, and for your earlier comments. We believe that the changes made based on your suggestions have greatly improved the detail and quality of the manuscript to address your concern. |
| Reviewer #3 | | |
| 19 | My comments are about the search methods.  The authors conducted a simple preliminary search to identify previously published reviews on the topic, in order to avoid duplication of effort and refine their topic. This search is well reported in the Supplementary materials. However, they also already performed what they refer to as the final search, in January and March 2021, in selected databases, with the assistance of a medical librarian; this search was peer-reviewed by a second medical librarian using the PRESS checklist. The search terms for each database are reported in the Supplementary materials and they look appropriate and well selected. Also, the reasons for using search limits (languages) are reported and explained.  I assume that the "full search" was run in order to select the appropriate search terms for each different database and show them in the protocol, but my concern is that the full search should not have been already run and reported in the protocol. By the time the protocol is published the search will need to be repeated to bridge the gap in the results, and for the general reader it is confusing to read of the search in the past tense when all the rest of the protocol is in the future tense. It is not necessary to see the number of results for each search line when they will be different once the search is re-run. Another reason not to perform the searches before the protocol is accepted for publication is that it is always possible that referees will suggest some changes to the search terms, or even that in the meantime there will be changes to the controlled vocabulary (such as Mesh) terms. I suggest to remove mentions of the full search in this protocol, report the search strategies for each database without the number of results for each line, and run the full searches once the protocol is published. | Thank you for the careful review and reflection of our work.  Thank you for raising this important concern; we agree, the search strategy should be presented in the future tense, given that this is a protocol and that we are not reporting results. To address your suggestion, we made the following adjustments to the manuscript and supplementary materials: (1) we removed the yield numbers and date of searches from the search strategies; (2) we removed references to the search having been completed (and changed all language to future tense), including the “Protocol registration and timeline” section, which had previously outlined which step(s) have already been completed. We will instead report this information in the final review papers to better help guide the reader. |
